# Nonsynonymous Mutations in *fepR* Are Associated with Adaptation of Listeria monocytogenes and Other *Listeria* spp. to Low Concentrations of Benzalkonium Chloride but Do Not Increase Survival of L. monocytogenes and Other *Listeria* spp. after Exposure to Benzalkonium Chloride Concentrations Recommended for Use in Food Processing Environments

**DOI:** 10.1128/aem.00486-22

**Published:** 2022-05-19

**Authors:** Samantha Bolten, Anna Sophia Harrand, Jordan Skeens, Martin Wiedmann

**Affiliations:** a Department of Food Science, Cornell University, Ithaca, New York, USA; University of Helsinki

**Keywords:** *Listeria monocytogenes*, *Listeria* spp., adaptation, benzalkonium chloride, survival, tolerance

## Abstract

Selection for Listeria monocytogenes strains that are tolerant to quaternary ammonium compounds (such as benzalkonium chloride [BC]) is a concern across the food industry, including in fresh produce processing environments. This study evaluated the ability of 67 strains of produce-associated L. monocytogenes and other *Listeria* spp. (“parent strains”) to show enhanced BC tolerance after serial passaging in increasing BC concentrations and to maintain this tolerance after substreaking in the absence of BC. After serial passaging in BC, 62/67 “BC passaged cultures” showed higher MICs (4 to 20 mg/L) than parent strains (2 to 6 mg/L). After the substreaking of two isolates from BC passaged cultures for each parent strain, 105/134 “adapted isolates” maintained MICs (4 to 6 mg/L) higher than parent strain MICs. These results suggested that adapted isolates acquired heritable adaptations that confer BC tolerance. Whole-genome sequencing and Sanger sequencing of *fepR,* a local repressor of the MATE family efflux pump FepA, identified nonsynonymous *fepR* mutations in 48/67 adapted isolates. The mean inactivation of adapted isolates after exposure to use-level concentrations of BC (300 mg/L) was 4.48 log, which was not significantly different from inactivation observed in parent strains. Serial passaging of cocultures of L. monocytogenes strains containing *bcrABC* or *qacH* did not yield adapted isolates that showed enhanced BC tolerance in comparison to that of monocultures. These results suggest that horizontal gene transfer either did not occur or did not yield isolates with enhanced BC tolerance. Overall, this study provides new insights into selection of BC tolerance among L. monocytogenes and other *Listeria* spp.

**IMPORTANCE** Listeria monocytogenes tolerance to quaternary ammonium compounds has been raised as a concern with regard to L. monocytogenes persistence in food processing environments, including in fresh produce packing and processing environments. Persistence of L. monocytogenes can increase the risk of product contamination, food recalls, and foodborne illness outbreaks. Our study shows that strains of L. monocytogenes and other *Listeria* spp. can acquire heritable adaptations that confer enhanced tolerance to low concentrations of benzalkonium chloride, but these adaptations do not increase survival of L. monocytogenes and other *Listeria* spp. when exposed to concentrations of benzalkonium chloride used for food contact surface sanitation (300 mg/L). Overall, these findings suggest that the emergence of benzalkonium chloride-tolerant *Listeria* strains in food processing environments is of limited concern, as even strains adapted to gain higher MICs *in vitro* maintain full sensitivity to the concentrations of benzalkonium chloride used for food contact surface sanitation.

## INTRODUCTION

Listeria monocytogenes is a foodborne pathogen that causes approximately 1,600 cases of foodborne illness and 260 deaths per year in the United States (1). L. monocytogenes persistence in built environments used for food production can provide a source of contamination in food products, which increases the risk of recalls and foodborne illness outbreaks (2, 3). While L. monocytogenes outbreaks and recalls have been linked to a diversity of foods, including dairy, seafood, and ready-to-eat deli meat (4), more recently (i.e., since 2009), L. monocytogenes has been implicated in several outbreaks related to environmental contamination in fresh produce packing or processing environments, including outbreaks associated with celery (5), cantaloupe (6), caramel apples (7), and packaged lettuce (8).

*Listeria* spp. (defined here as all species of *Listeria* excluding L. monocytogenes) are often referred to as index organisms for L. monocytogenes, and thus detection of *Listeria* spp. in a given processing facility location indicates conditions that would facilitate the presence and/or survival of L. monocytogenes in that same environment (9, 10). Throughout the world, regular monitoring of food production environments involves testing for all species of *Listeria* (including both *Listeria* spp. and L. monocytogenes), rather than L. monocytogenes alone (11). Therefore, it is important to understand how *Listeria* spp., in addition to L. monocytogenes, respond to interventions that seek to control L. monocytogenes.

Effective sanitation in food processing environments, including fresh produce packing and processing facilities, is essential for mitigating L. monocytogenes contamination of finished products. Quaternary ammonium compounds are sanitizers that are widely used for sanitation on both food contact and nonfood contact surfaces to control microorganisms such as L. monocytogenes and *Listeria* spp. (12). A commonly used class of quaternary ammonium compounds are alkyl-(C_8_-C_18_) dimethyl benzyl ammonium chlorides (also known as benzalkonium chlorides). Commercial formulations of sanitizers with benzalkonium chloride (BC) as an active ingredient are approved for use on food contact surfaces, utilized for nonorganic production, at concentrations between 150 and 400 mg/L (termed herein as use-level concentrations) in U.S. food packing, processing, and manufacturing environments (21 CFR 178.1010 [13]) (14). The efficacy of BC is measured by its ability to reduce target microorganism levels in a defined period (e.g., a 5-log reduction within 30 s) at use-level concentrations (15, 16). There is concern that frequent use of BC across the food industry, including in fresh produce packing and processing environments, may be contributing to the selection of L. monocytogenes and *Listeria* spp. that are less effectively inactivated by BC, which could increase the risk of persistence of L. monocytogenes and *Listeria* spp. in food facilities (17, 18).

Genetically encoded efflux pump systems represent an important mechanism of BC tolerance in L. monocytogenes and *Listeria* spp. (19). These efflux pumps can be encoded chromosomally, such as by the *fepRA* operon, which encodes the MATE family efflux pump FepA (20), or on mobile genetic elements, such as the three-gene cassette *bcrABC*, which is often found located on a pLM80-type plasmid, (21, 22), and *qacH*, which is located on the Tn*6188* transposon (23). Both *bcrABC* and *qacH* have been frequently observed in strains of L. monocytogenes and *Listeria* spp. isolated from food production environments and are known to confer tolerance of L. monocytogenes and *Listeria* spp. to BC (17, 24–28). There is concern that horizontal gene transfer of these genes could facilitate widespread and enhanced tolerance of L. monocytogenes and *Listeria* spp. to BC in the processing environment (29). Recently, an experimental study showed horizontal gene transfer of a mobile genetic element containing *bcrABC* as well as cadmium resistance genes from *Listeria* spp. to a streptomycin-resistant L. monocytogenes (30). In these experiments, selection for streptomycin resistance and cadmium resistance was used to obtain transformants (30), which can be argued to not be representative of the type of selection that is expected to occur in food-associated environments. Therefore, there is a need for studies that evaluate the horizontal gene transfer of BC resistance genes, and subsequent impacts on BC tolerance, in L. monocytogenes and *Listeria* spp. under conditions that more closely simulate real-world conditions.

There is considerable debate surrounding the terminology used to describe the ability of L. monocytogenes and *Listeria* spp. to grow in the presence of sanitizers like BC. With respect to clinical antibiotics, the term resistance is generally referred to, in both scientific literature (31) and by government organizations such as the World Health Organization (https://www.who.int/news-room/fact-sheets/detail/antimicrobial-resistance), as the ability of bacteria to maintain growth in therapeutic levels of an antibiotic agent. This definition is less relevant when applied to sanitizers such as BC, because use-level concentrations of BC are highly unlikely to support bacterial growth (32). In addition, in the context of food processing environments, improper development or implementation of sanitation standard operating procedures can result in the presence of BC at concentrations below use-level concentrations (18). Therefore, it is important to evaluate the growth of L. monocytogenes and *Listeria* spp. exposed to BC at concentrations below the recommended use-level concentrations. Recently, the term “tolerance” has been proposed as an alternative to the term “resistance” to describe the ability of microorganisms to show reduced susceptibility to sanitizers at concentrations below use-level concentrations (32). In this study, we sought to investigate the ability of L. monocytogenes and *Listeria* spp. to grow in low levels of BC (defined here as <20 mg/L), and we will henceforth use the term “tolerance” (as defined in reference 32) to refer to the capacity of strains of L. monocytogenes and *Listeria* spp. to acquire the ability to grow in low levels of BC to which they were once sensitive.

The specific purpose of this study was to investigate (i) the ability of monocultures of L. monocytogenes and *Listeria* spp. to acquire enhanced tolerance when exposed to sequentially increasing low levels of BC and (ii) the impact of this acquired enhanced BC tolerance on the ability of L. monocytogenes and *Listeria* spp. to survive exposure to use-level concentrations of BC. Additionally, we also examined (iii) whether L. monocytogenes cocultures that include strains carrying different BC resistance genes (i.e., *bcrABC*, *qacH*) could, after exposure to increasing BC concentrations, give rise to isolates with BC tolerance that exceeds tolerance observed in monocultures. Due to the recent emerging concerns about L. monocytogenes in fresh produce, we used produce-associated isolates for this study. However, as sanitizers such as BC are widely used for sanitation throughout food processing sectors, our findings should be applicable broadly to all food processing sectors.

## RESULTS

### Parent strains showed MICs to BC ranging from 1 to 6 mg/L, with strains carrying BC resistance genes showing significantly higher MICs.

**FIG 1:**
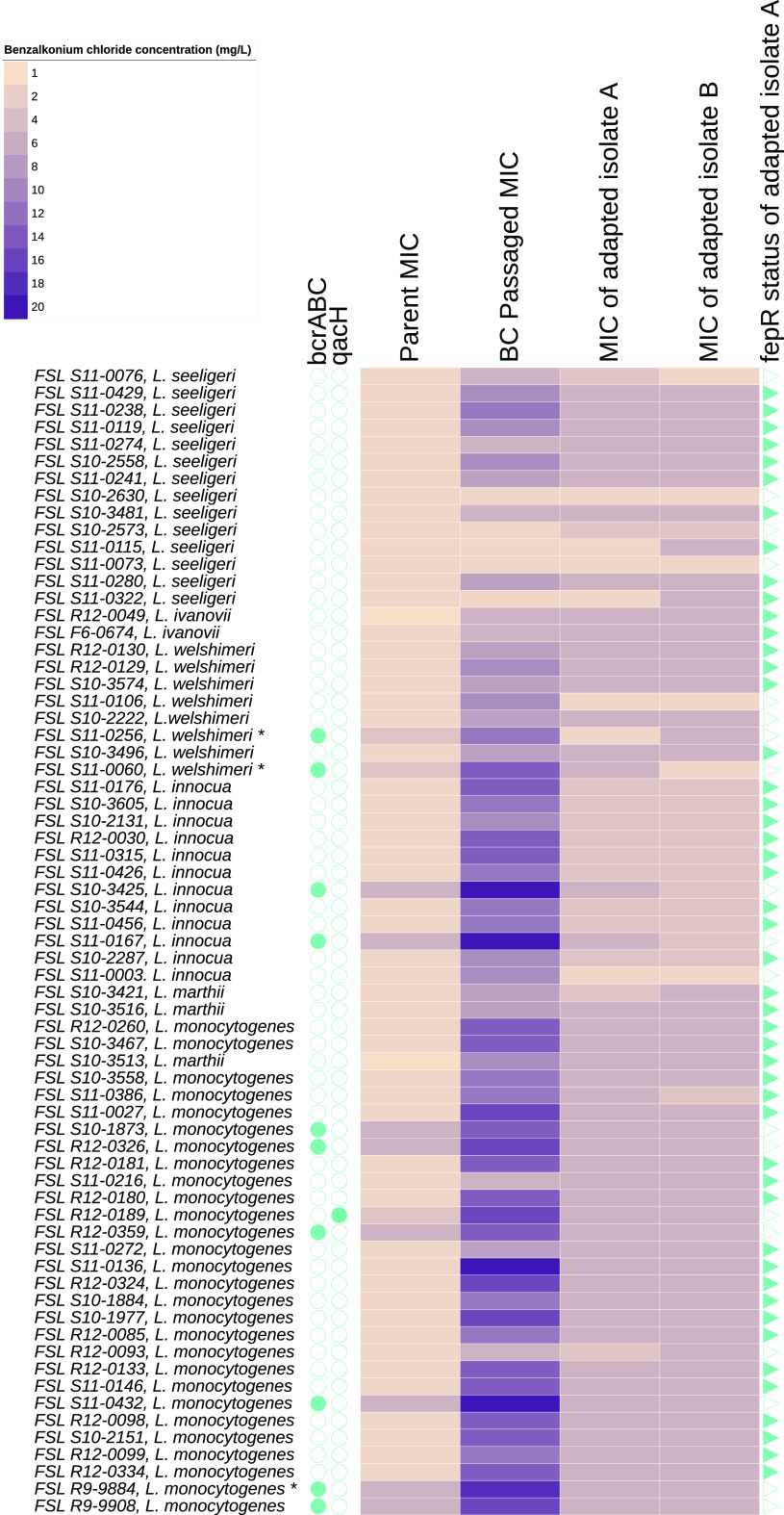
Heat map of *Listeria* MICs to BC. Each row is specific to an individual parent strain of *Listeria*. For example, row 1 of the heat map corresponds to strain FSL S11-0076, which is a strain of *L. seeligeri.* “Parent MIC” indicates the initial MIC obtained for a given *Listeria* strain; “BC passaged MIC” indicates the MIC obtained through serial passaging of a strain in increasing concentrations of BC; “MIC of adapted isolate A” and “MIC of adapted isolate B” indicate the MIC obtained from two isolates obtained from serial passage experiments that were substreaked seven times onto BHI agar. Blue filled circles represent the presence of a BC resistance gene (*bcrABC*, *qacH*) in the *Listeria* parent strain. FSL S11-0256 and FSL S11-0060 showed loss of *bcrABC* in their corresponding adapted isolate A, and FSL S11-0256 and FSL R9-9884 showed loss of *bcrABC* in their corresponding adapted isolate B (an asterisk is used to identify these parent strains that showed loss of *bcrABC* in one of their respective adapted isolates). Blue filled triangles represent the presence of a nonsynonymous mutation in *fepR* in adapted isolate A.

Initial MIC experiments of the 67 strains of L. monocytogenes and *Listeria* spp. characterized here (Table 1) showed a narrow range of MICs, from 1 to 6 mg/L, with an estimated marginal mean of 2.30 mg/L (Fig. 1); these initial MICs are referred to here as “parent MICs.” Results from two-way analysis of variance (ANOVA) and *post hoc* tests (Table 2) showed that strains of L. monocytogenes and *Listeria* spp. that carry the BC resistance genes *bcrABC* (*n* = 10) or *qacH* (*n* = 1) showed significantly higher MICs (estimated marginal mean of 5.37 mg/L; range of 4 to 6 mg/L BC) than strains lacking these genes (estimated marginal mean MIC of 1.95 mg/L; range of 1 to 2 mg/L BC) (*P* < 0.05). *bcrABC* was present in six L. monocytogenes, two *L. innocua*, and two *L. welshimeri* strains; *qacH* was present in one L. monocytogenes strain. Parent MICs for L. monocytogenes and *Listeria* spp. (i.e., all *L. innocua*, *L. ivanovii*, *L. marthii*, *L. seeligeri*, *and L. welshimeri* strains examined in this study) were not significantly different from one another, with an estimated marginal mean MIC for L. monocytogenes of 2.59 mg/L (range of 2 to 6 mg/L) and an estimated marginal mean MIC for *Listeria* spp. of 2.12 mg/L (range of 1 to 6 mg/L) (*P* > 0.05).

**TABLE 1:** Isolates of L. monocytogenes and *Listeria* spp. selected for BC susceptibility experiments

Parent strain FSL ID^*a*^	Adapted isolate A FSL ID^*b*^	Species	Isolation source (sample type)	Lineage	Clonal group^*c*^	Resistance gene^*d*^
FSL S11-0167	FSL H9-0106	*L. innocua*	Postharvest (environmental swab)	—^*e*^	—	*bcrABC*
FSL S10-3425	FSL H9-0109	*L. innocua*	Postharvest (environmental swab)	—	—	*bcrABC*
FSL S11-0456	FSL H9-0107	*L. innocua*	Postharvest (environmental swab)	—	—	NP
FSL S11-0003	FSL H9-0108	*L. innocua*	Postharvest (environmental swab)	—	—	NP
FSL S10-2287	FSL H9-0110	*L. innocua*	Preharvest (water)	—	—	NP
FSL S11-0176	FSL H9-0111	*L. innocua*	Postharvest (environmental swab)	—	—	NP
FSL S11-0315	FSL H9-0112	*L. innocua*	Postharvest (environmental swab)	—	—	NP
FSL S10-3544	FSL H9-0113	*L. innocua*	Postharvest (environmental swab)	—	—	NP
FSL S10-3605	FSL H9-0114	*L. innocua*	Postharvest (environmental swab)	—	—	NP
FSL S10-2131	FSL H9-0115	*L. innocua*	Preharvest (fecal)	—	—	NP
FSL S11-0426	FSL H9-0116	*L. innocua*	Postharvest (environmental swab)	—	—	NP
FSL R12-0030	FSL H9-0117	*L. innocua*	Postharvest (food)	—	—	NP
FSL S11-0073	FSL H9-0118	*L. seeligeri*	Postharvest (environmental swab)	—	—	NP
FSL S11-0115	FSL H9-0119	*L. seeligeri*	Postharvest (environmental swab)	—	—	NP
FSL S11-0119	FSL H9-0120	*L. seeligeri*	Postharvest (environmental swab)	—	—	NP
FSL S10-2573	FSL H9-0121	*L. seeligeri*	Preharvest (soil)	—	—	NP
FSL S10-2558	FSL H9-0122	*L. seeligeri*	Preharvest (soil)	—	—	NP
FSL S10-2630	FSL H9-0123	*L. seeligeri*	Postharvest (soil)	—	—	NP
FSL S11-0322	FSL H9-0124	*L. seeligeri*	Postharvest (environmental swab)	—	—	NP
FSL S11-3481	FSL H9-0125	*L. seeligeri*	Postharvest (environmental swab)	—	—	NP
FSL S11-0238	FSL H9-0126	*L. seeligeri*	Postharvest (environmental swab)	—	—	NP
FSL S11-0429	FSL H9-0127	*L. seeligeri*	Postharvest (environmental swab)	—	—	NP
FSL S11-0280	FSL H9-0128	*L. seeligeri*	Postharvest (environmental swab)	—	—	NP
FSL S11-0241	FSL H9-0129	*L. seeligeri*	Postharvest (environmental swab)	—	—	NP
FSL S11-0076	FSL H9-0130	*L. seeligeri*	Postharvest (environmental swab)	—	—	NP
FSL S11-0274	FSL H9-0131	*L. seeligeri*	Postharvest (environmental swab)	—	—	NP
FSL S10-3513	FSL H9-0132	*L. marthii*	Postharvest (environmental swab)	—	—	NP
FSL S10-3421	FSL H9-0133	*L. marthii*	Postharvest (environmental swab)	—	—	NP
FSL S10-3516	FSL H9-0134	*L. marthii*	Postharvest (environmental swab)	—	—	NP
FSL S11-0060	FSL H9-0135	*L. welshimeri*	Postharvest (environmental swab)	—	—	*bcrABC*
FSL S11-0106	FSL H9-0136	*L. welshimeri*	Postharvest (environmental swab)	—	—	NP
FSL R12-0129	FSL H9-0137	*L. welshimeri*	Postharvest (food)	—	—	NP
FSL R12-0130	FSL H9-0138	*L. welshimeri*	Postharvest (food)	—	—	NP
FSL S10-3574	FSL H9-0139	*L. welshimeri*	Postharvest (environmental swab)	—	—	NP
FSL S11-0256	FSL H9-0140	*L. welshimeri*	Postharvest (environmental swab)	—	—	*bcrABC*
FSL S10-2222	FSL H9-0142	*L. welshimeri*	Preharvest (soil)	—	—	NP
FSL S10-3496	FSL H9-0143	*L. welshimeri*	Postharvest (environmental swab)	—	—	NP
FSL F6-0674	FSL H9-0144	*L. ivanovii*	Postharvest (food)	—	—	NP
FSL R12-0049	FSL H9-0145	*L. ivanovii*	Postharvest (food)	—	—	NP
FSL R12-0334	FSL H9-0078	L. monocytogenes	Retail (environmental swab)	II	CC9	NP
FSL R9-9908	FSL H9-0079	L. monocytogenes	Postharvest (environmental swab)	II	CC9	*bcrABC*
FSL R12-0099^*f*^	FSL H9-0080	L. monocytogenes	Postharvest (environmental swab)	II	CC9	NP
FSL R12-0359	FSL H9-0081	L. monocytogenes	Retail (environmental swab)	I	CC5	*bcrABC*
FSL R12-0180	FSL H9-0082	L. monocytogenes	Retail (environmental swab)	II	CC193	NP
FSL R12-0326	FSL H9-0083	L. monocytogenes	Retail (environmental swab)	I	CC5	*bcrABC*
FSL R12-0181	FSL H9-0084	L. monocytogenes	Retail (environmental swab)	II	CC29	NP
FSL S10-1873	FSL H9-0085	L. monocytogenes	Preharvest (soil)	I	CC6	*bcrABC*
FSL S11-0386	FSL H9-0086	L. monocytogenes	Postharvest (environmental swab)	I	CC6	NP
FSL S11-0136	FSL H9-0087	L. monocytogenes	Postharvest (environmental swab)	I	CC4	NP
FSL S11-0272	FSL H9-0088	L. monocytogenes	Postharvest (environmental swab)	I	CC4	NP
FSL S10-1884	FSL H9-0089	L. monocytogenes	Preharvest (soil)	II	CC369	NP
FSL S10-1977	FSL H9-0090	L. monocytogenes	Preharvest (food)	II	CC369	NP
FSL S11-0146	FSL H9-0091	L. monocytogenes	Postharvest (environmental swab)	II	CC37	NP
FSL R12-0133	FSL H9-0092	L. monocytogenes	Retail (environmental swab)	II	CC37	NP
FSL S11-0216	FSL H9-0093	L. monocytogenes	Postharvest (environmental swab)	I	CC388	NP
FSL S11-0432	FSL H9-0094	L. monocytogenes	Postharvest (environmental swab)	II	CC155	*bcrABC*
FSL R12-0098	FSL H9-0095	L. monocytogenes	Postharvest (environmental swab)	II	CC155	NP
FSL S10-3467	FSL H9-0096	L. monocytogenes	Postharvest (environmental swab)	III	CC1789	NP
FSL R12-0260	FSL H9-0097	L. monocytogenes	Retail (environmental swab)	III	CC268	NP
FSL S10-3558	FSL H9-0098	L. monocytogenes	Postharvest (environmental swab)	III	CC434	NP
FSL R12-0093	FSL H9-0099	L. monocytogenes	Postharvest (environmental swab)	II	ST1861	NP
FSL S10-2151	FSL H9-0100	L. monocytogenes	Preharvest (soil)	II	CC204	NP
FSL R12-0085	FSL H9-0101	L. monocytogenes	Postharvest (environmental swab)	II	CC19	NP
FSL S11-0027	FSL H9-0102	L. monocytogenes	Postharvest (environmental swab)	I	CC6	NP
FSL R9-9884	FSL H9-0103	L. monocytogenes	Postharvest (environmental swab)	II	CC9	*bcrABC*
FSL R12-0189	FSL H9-0104	L. monocytogenes	Retail (environmental swab)	I	CC5	*qacH*
FSL R12-0324	FSL H9-0105	L. monocytogenes	Retail (environmental swab)	I	CC4	NP

a Isolates from Cornell Food Safety Lab (FSL) culture collection. Isolate information can be found on the Food Microbe Tracker website, https://www.foodmicrobetracker.net/login/login.aspx.

b Derivative isolates of parent strains that were obtained through serial passaging of parent strains in BC, followed by substreaking seven times in BC-free medium.

c Includes both clonal complexes (CCs) and singleton sequencing types (STs).

d These data indicate whether a parent strain possessed either *bcrABC* or *qacH* (genes associated with *Listeria* resistance to BC) or neither gene (NP, not present).

e —, not applicable, as only L. monocytogenes can be classified to lineage and clonal group using a standardized nomenclature.

f Strain FSL R12-0099 was derived from CFSAN007516 (SRA accession no. SRR1101447) which has a genome size of 3,023,201 bp and shows presence of the gene *bcrABC*. FSL R12-0099 in the Cornell FSL culture collection has a genome size of 2,947,467 bp and does not possess *bcrABC* (SRA accession no. SRR15829693). These two genomes differ in size by 75,734 bp, which is similar to the size of plasmid pLM80 (80,000 bp) on which the *bcrABC* cassette can be encoded (21). These results suggest evidence of the loss of *bcrABC* in FSL R12-0099.

**TABLE 2:** Marginal mean estimates for MICs based on interaction models I and II

Model and level	MIC estimate (mg/L)	SE	Lower CL^*a*^	Upper CL	Group^*b*^
Interaction model I^*c*^ (fixed effects interaction of MIC type–*Listeria* type)					
Parent MIC–L. monocytogenes	2.59	0.19	2.22	2.97	A
Parent MIC–*Listeria* spp.	2.12	0.13	1.86	2.37	A
BC passaged MIC–L. monocytogenes	13.38	0.98	11.45	15.31	E
BC passaged MIC–*Listeria* spp.	8.03	0.50	7.05	9.01	D
Adapted MIC–L. monocytogenes	5.91	0.34	5.25	6.58	C
Adapted MIC–*Listeria* spp.	4.41	0.21	3.99	4.83	B
					
Interaction model II^*d*^ (fixed effects interaction of MIC type–resistance gene)					
Parent MIC–present	5.37	0.59	4.21	6.53	B
Parent MIC–absent	1.95	0.10	1.76	2.14	A
BC passaged MIC–present	16.14	1.77	12.66	19.62	D
BC passaged MIC–absent	9.04	0.44	8.17	9.90	C
Adapted MIC–present	5.23	0.46	4.32	6.14	B
Adapted MIC–absent	4.94	0.19	4.56	5.32	B

a CL, confidence limit.

b Group refers to significant differences based on *post hoc* multiple-comparison adjustment with Tukey’s honestly significant difference (HSD) test. Within each interaction model (I or II), groups that do not share a given letter are significantly different (*P* < 0.05).

c Interaction model I tests the interaction of fixed effects of (i) *Listeria* type (either L. monocytogenes or *Listeria* spp. [*L. innocua*, *L. ivanovii*, *L. marthii*, *L. seeligeri*, *L welshimeri*]) and (ii) MIC type (either parent MIC, BC passaged MIC, or adapted MIC) on MIC to BC. Parent MIC, the initial MIC for a strain of L. monocytogenes or *Listeria* spp.; BC passaged MIC, the MIC obtained through serial passaging a strain in increasing concentrations of BC; adapted MIC, the MIC obtained from two isolates obtained from serial passage experiments that were substreaked seven times onto BHI agar.

d Interaction model II tests the interaction of fixed effects of (i) resistance gene present (strains of L. monocytogenes or *Listeria* spp. that carry *bcrABC* or *qacH*) or absent (strains of L. monocytogenes or *Listeria* spp. that do not carry *bcrABC* or *qacH*) and (ii) MIC type (either parent MIC, BC passaged MIC, or adapted MIC) on MICs for BC.

### Serial passaging experiments showed that 62/67 parent strains were able to grow in BC concentrations above their parent MIC.

To assess the ability of L. monocytogenes and *Listeria* spp. to acquire enhanced tolerance to BC, monocultures of all 67 parent strains of L. monocytogenes and *Listeria* spp. were subjected to serial passaging in increasing concentrations of BC. The highest concentration of BC in which a given strain failed to show growth after 48 h of incubation was designated the “BC passaged MIC.” The estimated marginal mean BC passaged MIC for all 67 strains was 9.94 mg/L, which was significantly higher than the estimated marginal mean parent MIC of 2.30 mg/L (*P* < 0.05). Overall, 62/67 strains were able to grow to BC concentrations above their parent MICs. For these strains, BC passaged MICs ranged from 4 to 20 mg/L. The five strains that did not grow above parent MICs all belonged to the species *L. seeligeri*. Results from two-way ANOVA and *post hoc* tests showed that the BC passaged MICs for L. monocytogenes (estimated marginal mean of 13.38 mg/L; range of 6 to 20 mg/L) were significantly higher (*P* < 0.05) than the BC passaged MICs for *Listeria* spp. (estimated marginal mean of 8.03 mg/L; range of 2 to 20 mg/L) (Table 2).

To further characterize the impact of serial passages in the presence of BC on MICs, we also calculated the MIC fold change for all strains (Table 3). The fold change of BC passaged MIC/parent MIC for all strains ranged from 1 to 10. L. monocytogenes showed a slightly higher fold change (estimated marginal mean fold change of 4.74) than *Listeria* spp. (estimated marginal mean fold change of 4.03), but this difference was not significant (*P* > 0.05). The fold change of the BC passaged MIC relative to the parent MIC (BC passaged MIC/parent MIC) for strains of L. monocytogenes and *Listeria* spp. that carried BC resistance genes *bcrABC* or *qacH* was significantly lower (estimated marginal mean fold change of 2.25) than the fold change observed in strains that did not carry these BC resistance genes (estimated marginal mean fold change of 4.90) (*P* < 0.05).

**TABLE 3:** Estimates of marginal means for fold change of MICs based on additive models I and II

Model	Fixed effects	Level	Estimated fold change	SE	Lower CL^*a*^	Upper CL	Group^*b*^
I^*c*^	Fold change type–*Listeria* type	BC passaged MIC/parent MIC–L. monocytogenes	4.74	0.43	3.89	5.60	B
		BC passaged MIC/parent MIC–*Listeria* spp.	4.03	0.32	3.40	4.66	B
		Adapted MIC/parent MIC–L. monocytogenes	2.38	0.21	1.97	2.79	A
		Adapted MIC/parent MIC–*Listeria* spp.	2.02	0.15	1.73	2.32	A
							
II^*d*^	Fold change type–resistance gene	BC passaged MIC/parent MIC–present	2.25	0.25	1.76	2.74	B
		BC passaged MIC/parent MIC–absent	4.90	0.28	4.35	5.46	C
		Adapted MIC/parent MIC–present	1.13	0.12	0.89	1.36	A
		Adapted MIC/parent MIC–absent	2.46	0.12	2.22	2.70	B

a CL, confidence limit.

b Group refers to significant differences based on *post hoc* multiple-comparison adjustment with Tukey’s honestly significant difference (HSD) test. Within each additive model (I or II), different numbers denote significant differences (*P* < 0.05).

c Additive model I tests the fixed effects of (i) *Listeria* type (either L. monocytogenes or *Listeria* spp. [*L. innocua*, *L. ivanovii*, *L. marthii*, *L. seeligeri*, *L welshimeri*]) and (ii) MICs being compared (BC passaged MIC/parent MIC, adapted MIC/parent MIC) on the level of fold change. Parent MIC, the initial MIC for a strain of L. monocytogenes or *Listeria* spp.; BC passaged MIC, the MIC obtained through serial passaging a strain in increasing concentrations of BC; adapted MIC, the MIC obtained from two isolates obtained from serial passage experiments that were substreaked seven times onto BHI agar.

d Additive model II tests the fixed effects of (i) resistance gene present (strains of L. monocytogenes or *Listeria* spp. that carry *bcrABC* or *qacH*) or absent (strains of L. monocytogenes or *Listeria* spp. that do not carry *bcrABC* or *qacH*) and (ii) MICs being compared (BC passaged MIC/parent MIC, adapted MIC/parent MIC) on the level of fold change.

### L. monocytogenes and *Listeria* spp. maintain increased MICs after seven rounds of substreaking in the absence of BC.

Following serial passaging experiments, the culture that represented the concentration of brain heart infusion (BHI) broth supplemented with BC (BHI-BC) in which a given strain of L. monocytogenes or *Listeria* spp. failed to show growth was plated onto BC-free BHI agar (BHIA) and two individual colonies (adapted isolate A and adapted isolate B) were selected for further characterization. All colonies were substreaked for seven rounds on BC-free BHIA to determine whether the tolerance of L. monocytogenes and *Listeria* spp. to BC was maintained in the absence of selective pressure. These data were used to determine whether increased MICs acquired during serial passaging were due to transient or inherited tolerance. After seven rounds of substreaking, all isolates were characterized through MIC experiments, and the resulting MICs were designated “MIC of adapted isolate A” and “MIC of adapted isolate B” (Fig. 1). The estimated marginal mean adapted MIC for all L. monocytogenes and *Listeria* spp. was 4.99 mg/L (range of 2 to 6 mg/L), which was significantly higher than the estimated marginal mean parent MIC (2.30 mg/L) but also significantly lower than the estimated marginal mean BC passaged MIC (9.94 mg/L) (*P* < 0.05).

The majority of adapted isolates showed higher adapted MICs than their parent MICs (105/134) but lower adapted MICs than their BC passaged MICs (113/134). Results from two-way ANOVA and *post hoc* tests showed that L. monocytogenes adapted MICs were significantly higher (estimated marginal mean of 5.91 mg/L, range of 4 to 6 mg/L) than adapted MICs of *Listeria* spp. (estimated marginal mean of 4.41 mg/L, range of 2 to 6 mg/L) (*P* < 0.05). The presence (estimated marginal mean of 5.23 mg/L, range of 2 to 6 mg/L) or absence (estimated marginal mean of 4.94 mg/L, range of 2 to 6 mg/L) of BC resistance genes *bcrABC* or *qacH* was not associated with a significant difference in adapted MICs (*P* > 0.05) (Table 2).

Further analysis of the fold change of adapted MIC/parent MIC (Table 3) showed that the fold change of L. monocytogenes (estimated marginal mean fold change of 2.38) did not differ significantly from the fold change of *Listeria* spp. (estimated marginal mean fold change of 2.02) (*P* > 0.05), suggesting no difference in the abilities of L. monocytogenes and *Listeria* spp. to adapt to BC. The estimated marginal mean fold change of adapted MIC/parent MIC for L. monocytogenes and *Listeria* spp. that carried either *bcrABC* or *qacH* was significantly lower (estimated marginal mean fold change of 1.13) than the fold change of isolates that did not carry *bcrABC* or *qacH* (estimated marginal mean fold change of 2.46) (*P* < 0.05), suggesting that isolates carrying *bcrABC* or *qacH* showed limited adaptation to BC.

The 67 strains in this study were selected from a larger culture collection, where they were previously classified as the top 10% tolerant, top 10% sensitive, or average in their sensitivity (representing the 11th to 89th percentile) to use-level concentrations of BC (based on log reduction data collected in a previous study) (33). Hence, we examined whether these previous classifications for the strains of L. monocytogenes and *Listeria* spp. we selected were associated with the MICs for these strains. Our results found that the previous classification of strains of L. monocytogenes and *Listeria* spp. as tolerant, average, or sensitive to use levels of BC was not significantly associated with a difference in parent MICs, BC passaged MICs, or adapted MICs (*P* > 0.05).

### Mutations in *fepR* are associated with enhanced tolerance of L. monocytogenes and *Listeria* spp. to low levels of BC.

To investigate putative mutations responsible for the observed enhanced tolerance in adapted isolates, we performed whole-genome sequencing (WGS) on a subset of adapted isolate A isolates (*n *= 16) and compared their genomes to the genomes of their respective parent strains using high-quality single nucleotide polymorphism (hqSNP) analysis. In all 16 adapted isolates sequenced, we detected mutations in *fepR* (lmo2088), a gene encoding a TetR family transcriptional regulator (Table 4). Mutations detected through hqSNP analysis included (i) nonsense mutations (i.e., SNPs that lead to premature stop codons in the coding sequence of *fepR*) in 8/16 isolates and (ii) missense mutations (i.e., SNPs that lead to an amino acid change in the *fepR* coding sequence) in 5/16 isolates. In addition, single nucleotide deletions leading to frameshift mutations in *fepR* were detected in 3/16 isolates; these deletions were detected by aligning genome assemblies of parent strains and adapted isolates to an annotated *fepR* sequence. Other mutations detected in adapted isolates included a missense mutation in an internalin-like protein in adapted isolate FSL H9-0100 and a nonsense mutation detected in the putative membrane protein YdfK in adapted isolate FSL H9-0112.

**TABLE 4:** Mutations detected in *fepR* for adapted isolates of L. monocytogenes or *Listeria* spp.^*a*^

Adapted isolate ID	Species	Total no. of SNPs in adapted isolate	Mutation detected in *fepR*^*b*^	Mutation type^*c*^ in *fepR*	BioProject accession no. or SRA ID of parent strain^*d*^
FSL H9-0080	L. monocytogenes	0	Deletion	Frameshift	PRJNA761983
FSL H9-0082	L. monocytogenes	1	SNP	Missense	SRR9019233
FSL H9-0095	L. monocytogenes	2	SNP	Nonsense	SRR1027706
FSL H9-0098	L. monocytogenes	2	SNP	Missense	PRJNA761983
FSL H9-0100	L. monocytogenes	4	SNP	Nonsense	SRR12696480
FSL H9-0097	L. monocytogenes	1	SNP	Nonsense	SRR9019214
FSL H9-0105	L. monocytogenes	1	Deletion	Frameshift	SRR9019309
FSL H9-0111	*L. innocua*	2	SNP	Nonsense	PRJNA761983
FSL H9-0112	*L. innocua*	4	SNP	Nonsense	PRJNA761983
FSL H9-0122	*L. seeligeri*	1	SNP	Missense	PRJNA761983
FSL H9-0131	*L. seeligeri*	2	SNP	Nonsense	PRJNA761983
FSL H9-0132	*L. marthii*	2	SNP	Missense	PRJNA761983
FSL H9-0134	*L. marthii*	1	SNP	Missense	PRJNA761983
FSL H9-0137	*L. welshimeri*	0	Deletion	Frameshift	PRJNA761983
FSL H9-0144	*L. ivanovii*	1	SNP	Nonsense	PRJNA761983
FSL H9-0145	*L. ivanovii*	2	SNP	Nonsense	PRJNA761983

a Mutations were identified by comparison of the whole-genome sequences of parent strains and adapted isolates.

b *fepR* encodes a transcriptional regulator in the TetR family of transcriptional regulators (GenBank accession no. WP_010991061.1). SNP, single nucleotide polymorphism detected in *fepR*; deletion, deletion of a single nucleotide in *fepR.*

c Either missense (SNP confers amino acid shift in FepR protein), nonsense (either SNP confers a premature stop codon in FepR), or deletion (confers a frameshift in FepR).

d Whole-genome sequences of all adapted isolates can be found under BioProject accession number PRJNA761983. The parent strain ID that corresponds to each adapted isolate ID is shown in Table 1.

**FIG 2:**
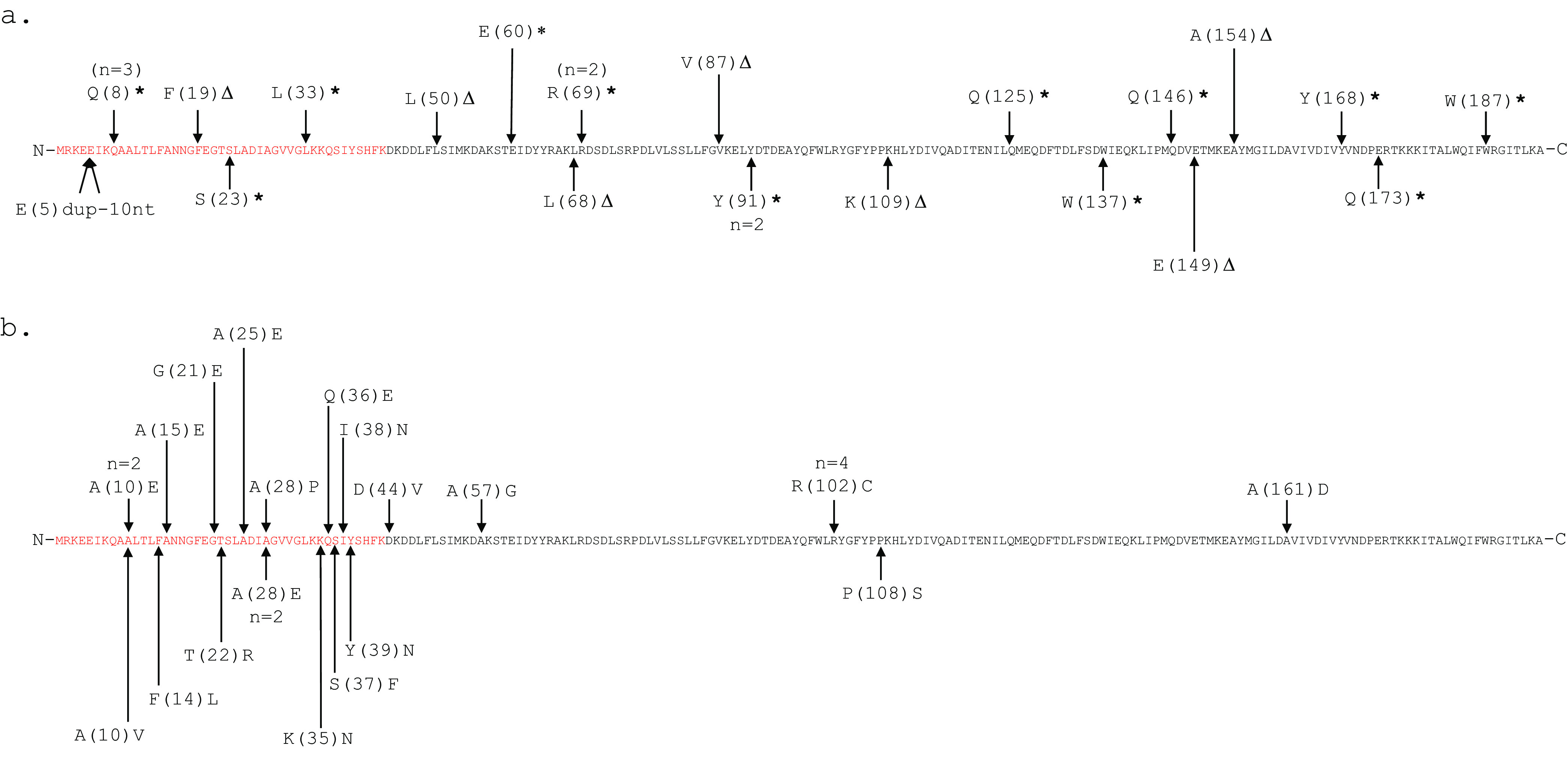
Location of nonsynonymous mutations in FepR in adapted isolates of L. monocytogenes and *Listeria* spp. from this study. Arrows indicate amino acid residues in which adapted isolates acquired unique mutations, and *n* is the number of isolates in which the unique mutation was detected. Amino acids colored in red represent the FepR DNA binding domain (45). (a) Nonsynonymous mutations that result in a nonsense mutation are denoted by an asterisk, single nucleotide deletions resulting in frameshift mutations are denoted by Δ, and a duplication resulting in a frameshift mutation (denoted by “dup-number of nucleotides” in length of duplication) (*n *= 24) are annotated on the 194-amino-acid FepR sequence (GenBank no. WP_010991061.1). (b) Nonsynonymous mutations that result in a missense mutation (*n *= 24) are annotated on the 194-amino-acid FepR sequence (GenBank no. WP_010991061.1).

On the basis of the high frequency of *fepR* mutations in adapted isolates, we PCR amplified the *fepR* sequence, followed by Sanger sequencing of PCR amplicons, for the 51 remaining adapted isolate A isolates that had not been characterized by WGS. A comparison of the *fepR* gene sequence in all 67 adapted isolates and parent strains revealed that 48/67 of the adapted isolates of L. monocytogenes and *Listeria* spp. in this study showed nonsynonymous mutations in *fepR* (24 missense mutations, 16 nonsense mutations, and 8 frameshift mutations) compared to their respective parent strains (Fig. 1). Among the frameshift mutations detected, seven were the result of single nucleotide deletions, and one was the result of a duplication of 10 nucleotides in *fepR* in adapted isolate FSL H9-0115 (Fig. 2). Among the missense mutations detected, 16/24 were localized in the N-terminal DNA binding domain of FepR between amino acid residues 1 and 43; five nonsense and two frameshift mutations were also detected in this region of FepR. None of the 11 parent strains that carried *bcrABC* or *qacH* acquired *fepR* mutations in their respective adapted isolates. ANOVA and *post hoc* tests revealed that the presence of a *fepR* mutation in adapted isolates was significantly associated with enhanced adapted MICs (estimated marginal mean MICs of 5.27 mg/L and 4.21 mg/L for isolates that possess *fepR* mutation and do not possess *fepR* mutation, respectively) (*P* < 0.05), while no significant differences in adapted MICs were detected across the three types of mutations detected in *fepR* (i.e., nonsense, missense, frameshift) (*P* > 0.05).

### While some adapted isolates show loss of *bcrABC*, this does not always result in reduced MICs.

PCR detection of *bcrABC* for adapted isolates revealed that four adapted isolates showed loss of *bcrABC* compared to their parent strains (including adapted isolates A and B derived from parent strain FSL S11-0256, adapted isolate A derived from parent strain FSL S11-0060, and adapted isolate B derived from parent strain FSL R9-9884) (Fig. 1). While two of these adapted isolates showed lower MICs than their respective parent strains, the other two adapted isolates (adapted isolate B from parent strain FSL S11-0256 and adapted isolate B from parent strain FSL R9-9884) both showed the same MICs as their respective parent strains (6 mg/L). In addition, both adapted isolate B from parent strain FSL S11-0256 and adapted isolate B from parent strain FSL R9-9884 did not show *fepR* mutations. These findings suggest possible additional mechanisms of adaptation to BC that could be further explored in future studies.

### Tolerance of L. monocytogenes and *Listeria* spp. to low levels of BC is not associated with increased survival at use-level concentrations of BC.

**FIG 3:**
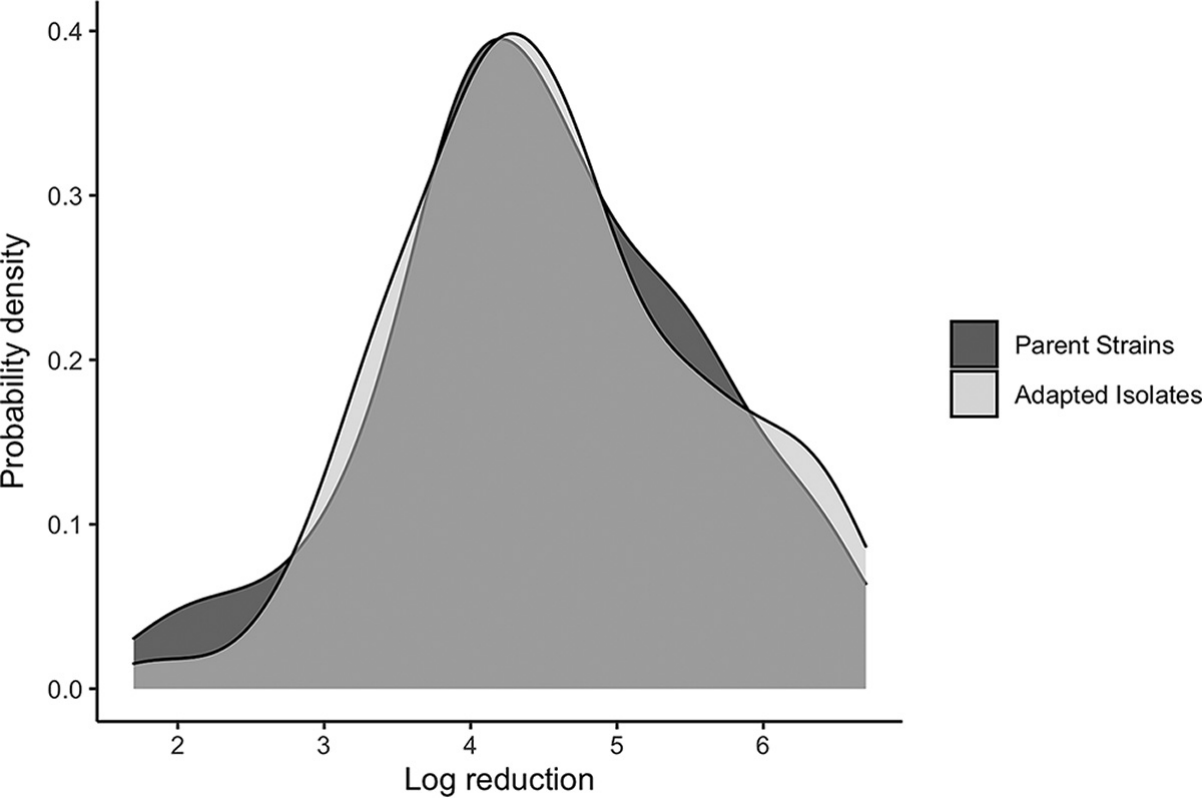
Density plot comparing log reductions of parent strains and adapted isolates after exposure to a use-level concentration (300 mg/L) of BC.

In addition to investigating the tolerance of L. monocytogenes and *Listeria* spp. to low levels of BC, all parent strains and adapted isolate A isolates were assessed for their survival when exposed to a use-level concentration of BC (300 mg/L) for 30 s. Initial populations of parent strains (mean of 8.46 ± 0.02 log CFU/mL) and adapted isolates (mean of 8.45 ± 0.02 log CFU/mL) were reduced by 1.7 to 6.7 log (mean of 4.56 ± 0.09) and 1.8 to 6.6 log (mean of 4.48 ± 0.09), respectively, after treatment with BC (Fig. 3). An unpaired *t* test showed that log reductions of parent strains and adapted isolates were not significantly different (*P* = 0.57). Furthermore, the 11 parent strains that contained *bcrABC* or *qacH* and the 56 parent strains that did not contain *bcrABC* or *qacH* also did not differ significantly (*P* = 0.65) in observed log reductions in the presence of BC (mean log reductions of 4.47 ± 0.21 and 4.57 ± 0.10, respectively) (raw data deposited in GitHub).

### Cocultures of L. monocytogenes strains did not yield adapted isolates that show higher BC tolerance than monocultures exposed to BC.

Cocultures of L. monocytogenes containing either *bcrABC* or *qacH*, and strains containing neither genetic resistance determinant, were initially cocultured on a 0.45-μm filter in the absence of BC and subsequently serially passaged in increasing concentrations of BC to select for BC tolerance. This was followed by substreaking in the absence of BC to select for adapted isolates with heritable enhanced BC tolerance. The goal of these experiments was to determine whether the combination of conditions that facilitate horizontal gene transfer and select for BC tolerance could give rise to adapted isolates that showed enhanced BC tolerance in comparison to adapted isolates obtained from monocultures of L. monocytogenes. MICs of cocultures and monoculture controls immediately after filter mating on filter plates ranged from 8 to 10 mg/L, except for the monoculture of strain FSL R12-0334 (the only monoculture strain that contained neither *bcrABC* nor *qacH*), which showed an MIC of 4 mg/L (Table 5). After serial passaging, all monocultures and cocultures achieved BC passaged MICs in the range of 10 to 14 mg/L; cocultures did not show significantly higher BC passaged MICs than monocultures (*P* > 0.05). Similarly, MIC experiments performed on adapted isolates obtained after seven rounds of substreaking from monoculture and coculture serial passage experiments revealed that the adapted MIC across all adapted isolates obtained from cocultures and monocultures was 6 mg/L. Thus, L. monocytogenes cocultures did not yield adapted isolates that showed enhanced tolerance to BC compared to monocultures. These findings suggest that no rearrangements of BC resistance genes (*bcrABC*, *qacH*) occurred that resulted in enhanced BC tolerance in adapted isolates. As none of the adapted isolates from L. monocytogenes cocultures showed higher tolerance than adapted isolates from L. monocytogenes monocultures, no further characterization (e.g., subtyping to determine the identity of the adapted isolates) was performed.

**TABLE 5:** MICs of BC for L. monocytogenes monocultures and cocultures obtained by three different experiments

Isolate ID (abbreviation)^*a*^	Lineage, resistance gene	MIC (mg/L) obtained by indicated assay^*b*^		
		Filter plate MIC	BC passaged MIC	Adapted MIC
Monoculture FSL S11-0432 (S1)	II, *bcrABC*	10	14	6
Monoculture FSL S10-1873 (S2)	II, *bcrABC*	10	14	6
Monoculture FSL R9-9884 (S3)	II, *bcrABC*	10	12	6
Monoculture FSL R12-0334 (S4)	II, none^*c*^	4	10	6
Monoculture FSL R12-0326 (S5)	I, *bcrABC*	8	14	6
Monoculture FSL R12-0189 (S6)	I, *qacH*	8	12	6
Monoculture FSL R12-0359 (S7)	I, *bcrABC*	10	14	6
				
Coculture I (S1, S3, S5, S6)		10	12	6
Coculture II (S1, S5, S6, S7)		10	12	6
Coculture III (S1, S4, S5, S6)		8	10	6
Coculture IV (S2, S5, S6, S7)		10	12	6
Coculture V (S2, S4, S5, S6)		10	12	6
Coculture VI (S2, S3, S5, S6)		10	14	6

a Monoculture, individual L. monocytogenes strains. Coculture, mixture of four L. monocytogenes strains combined and cultured together. Abbreviations in parentheses are isolate abbreviations used to describe the four isolates in each coculture.

b Filter plate MIC, results from an MIC assay performed on cultures obtained after either four L. monocytogenes strains (i.e., cocultures), or individual L. monocytogenes strains (i.e., monocultures), were placed onto a 0.45-μm filter on BHI agar plates and allowed to incubate at 22°C for 48 h. BC passaged MIC, MIC obtained through serial passaging of L. monocytogenes cocultures I to VI or of monocultures (S1 to S7) in increasing concentrations of BC. Adapted MIC, the MIC for three adapted isolates that were taken from enumeration plates after serial passage experiments and substreaked for seven rounds onto BHI agar. All three adapted isolates (adapted isolate A, adapted isolate B, adapted isolate C) displayed the same adapted MIC.

c No resistance gene (*bcrABC* or *qacH*) was identified in the genome.

## DISCUSSION

In this study, we assessed the capacity of 67 diverse produce-associated strains of L. monocytogenes and *Listeria* spp. to acquire tolerance to low levels of BC, a sanitizer commonly used in produce packing and processing environments, and evaluated whether acquired tolerance to low levels of BC was associated with increased survival of L. monocytogenes and *Listeria* spp. when exposed to use-level concentrations of BC (i.e., 300 mg/L). While all strains of L. monocytogenes and *Listeria* spp. used in this study were isolated from environments associated with fresh produce production, since sanitizers such as BC are used widely throughout the food industry, our findings presented here are applicable beyond fresh produce packing and processing environments.

### L. monocytogenes and *Listeria* spp. exposed to BC in serial passage experiments acquire inheritable and transient tolerance to low levels of BC.

In this study, we found that 105/134 adapted isolates of L. monocytogenes and *Listeria* spp. were able to acquire and maintain enhanced tolerance to BC through serial passage in increasing concentrations of BC and seven rounds of substreaking on BC-free media. By showing that this enhanced tolerance could be maintained in adapted isolates after selective pressure was removed, we have evidence to conclude that the acquired tolerance to BC in adapted isolates was due to inheritance of genetic mutations selected for during serial passaging. This phenomenon is referred to as tolerance due to genetic adaption (34). Additionally, we found that all species of *Listeria* represented in this study (*L. innocua*, *L. ivanovii*, *L. marthii*, L. monocytogenes, *L. seeligeri*, and *L. welshimeri*) could adapt to levels of BC up to 3-fold higher than their parent MICs. This is consistent with previous studies that have shown that BC-adapted L. monocytogenes strains can show 2- to 3-fold-higher MICs to BC than their wild-type counterparts (35, 36).

The majority of strains of L. monocytogenes and *Listeria* spp. were able to achieve higher BC passaged MICs than their parent MICs. However, for many strains of L. monocytogenes and *Listeria* spp., this acquired tolerance was not maintained or fully maintained after isolates were substreaked on BC-free medium. Thus, serial passage experiments also allowed L. monocytogenes and *Listeria* spp. to acquire transient tolerance to BC, which suggests that L. monocytogenes and *Listeria* spp. showed acclimation to BC (34) in addition to adaptation. Several studies have reported Gram-positive organisms acquiring transient tolerance to BC (37–39), even though some of these studies may not have referred to this observed phenomenon as “acclimation.” For example, in a study by Moore et al. (37), Staphylococcus haemolyticus was able to acquire tolerance to concentrations of BC that were 35-fold higher than its parental MIC (MIC value of 0.45 mg/L), but this tolerance was lost after passaging in the absence of BC for 7 days.

While horizonal gene transfer was previously reported to contribute to dispersal for *bcrABC* (30, 40), in our study, cocultures of L. monocytogenes strains carrying *bcrABC*, *qacH*, or neither genetic resistance determinant did not yield adapted isolates with enhanced tolerance to BC compared to the tolerance observed in adapted isolates obtained from monocultures. These findings could be because (i) selective pressure imposed by BC exposure in cocultures was not strong enough to select for transconjugants that did possess higher tolerance to BC or (ii) horizontal gene transfer of *bcrABC* and *qacH* did not occur within the coculture populations. This suggests that transfer of BC resistance genes to recipient isolates that already have resistance genes may be unlikely to occur or might not be strongly selected for, at least under the conditions used here. However, our study was limited to investigating the emergence of L. monocytogenes with enhanced BC tolerance among L. monocytogenes strains that were serially passaged in BC in planktonic cultures. As such, our findings may not be representative of interspecies and intergeneric transfer of BC resistance genes *bcrABC* and *qacH* and of selection that could occur in sessile cultures or multispecies biofilms. For example, interspecies transfer of *bcrABC* has been reported from *Listeria* spp. to L. monocytogenes (30), and intergeneric transfer of *bcrABC* has been reported from L. monocytogenes to Escherichia coli (40). As horizontal gene transfer continues to represent a potential mechanism for spread of BC tolerance in food processing environments (41, 42), future experiments using different approaches to select for BC-tolerant isolates should thus be performed to further probe whether horizontal gene transfer could give rise to transconjugants that do carry more than one BC resistance gene (i.e., *bcrABC* and *qacH*) and show an enhanced tolerance to BC compared to isolates that carry only one BC resistance gene.

Notably, our experiments revealed that L. monocytogenes and *Listeria* spp. (cocultures and monocultures) showed adaptation to a maximum MIC of 6 mg/L. Similar results were reported by Aase et al. (36), who showed that L. monocytogenes isolates with parent MICs to BC ranging from 1 to 7 mg/L were able to acquire inheritable tolerance to a maximum MIC of 7 mg/L BC. Results from Aase et al. (36) and our study both suggest that there is a biological barrier to the level of inheritable tolerance that L. monocytogenes and *Listeria* spp. can acquire to BC, with that level being ~6 to 7 mg/L. Most importantly, these findings support the view that there are limited concerns about L. monocytogenes and *Listeria* spp. acquiring tolerance to BC at levels that would impact the efficacy of quaternary ammonium compounds when they are used at recommended use-level concentrations.

### Acquired tolerance of L. monocytogenes and *Listeria* spp. to BC is associated with nonsynonymous mutations in *fepR*.

The majority of strains of L. monocytogenes and *Listeria* spp. in our study acquired enhanced tolerance to BC, and this tolerance was significantly associated with the presence of nonsynonymous mutations (including missense, nonsense, and frameshift) in *fepR*. Importantly, adapted isolates representing all *Listeria* species included in this study showed evidence of mutations that would abolish FepR function. FepR has previously been shown to act as a local repressor for transcription of the operon *fepRA* (20). In addition to self-regulation, FepR also represses the transcription of *fepA* (lmo2087), which encodes an efflux pump that is part of the multidrug and toxic compound extrusion (MATE) family (43), which conceivably could remove BC compounds from the bacterial cytosol.

Consistent with our findings, Guérin et al. (20) reported that a single nonsense mutation in *fepR* in L. monocytogenes strain BM4716 was associated with a 2-fold-higher MIC to BC than that of L. monocytogenes parent strain BM4715. Moreover, strain BM4716 and a *fepR* deletion mutant of BM4715 (BM4715Δ*fepR*) both showed a 64-fold increase in gene expression of FepA compared to BM4715, demonstrating that loss of function of FepR results in overexpression of FepA. However, MIC assays conducted in the presence of the efflux pump inhibitor reserpine did not lead to the expected reduction in MIC for strain BM4716 (20), which may suggest that overexpression of efflux pump FepA may not be responsible for the enhanced BC tolerance associated with *fepR* mutations. Alternatively, reserpine added at a concentration of 10 mg/L may not have been used at the appropriate concentration to inhibit a highly expressed FepA or may not inhibit FepA as effectively as it does other efflux pumps. This is consistent with observations by Meier et al. (27), who found that 10 mg/L reserpine did not inhibit efflux activity in all eight of the L. monocytogenes strains that carried *bcrABC* in their study. Overall, data available to date strongly support that mutations in *fepR* resulting in truncation and loss of function in FepR are associated with and responsible for enhanced tolerance to low levels of BC.

In addition to mutations resulting in truncation of FepR (nonsense and frameshift mutations), we also observed missense mutations in 24 adapted isolates of L. monocytogenes and *Listeria* spp., and 22 of those isolates also showed the phenotype of acquired enhanced tolerance to BC. Similar to our findings, Bland et al. (44) showed that L. monocytogenes can acquire similar levels of tolerance to BC through both nonsense and missense mutations in *fepR*. In our study, we detected the majority (16/24) of missense mutations in the N-terminal DNA binding domain (NDB; helices α1 to α3) of FepR, which makes up the helix-turn-helix (HTH) motif that binds to the operator sequence of DNA on the *fepRA* operon (45). This NDB region of FepR is highly conserved with other TetR family transcriptional regulators (45); hence, previous findings in FepR homologues may be used to elucidate the likely effect of these missense mutations on FepR function. For example, TetR family regulators QacR and TetR both show a lack of water present at the NDB-operator DNA sequence binding interface, which facilitates tight binding of their NDB regions to DNA operator sequences (46, 47). Here, we saw 8/16 mutations in the NDB region of FepR that showed an amino acid change to glutamic acid (E) and 3/16 mutations that showed an amino acid change to asparagine (N), both of which have polar side chains that can participate in hydrogen bonding. We hypothesize that the incorporation of E and N residues in FepR’s NDB allows for the incorporation of water into the NDB-operator DNA sequence binding interface of *fepRA*, which can cause reduced or inhibited binding of FepR to the *fepRA* operator.

In addition, in our study, we also observed a handful of missense mutations located in regions localized outside of FepR’s NDB. One such mutation that was observed in *L. innocua* FSL H9-0107, an adapted isolate that showed 2-fold-enhanced tolerance to BC compared to its parent strain, resulted in an amino acid change from proline to serine at residue 108. Notably, this same amino acid change was reported by Bland et al. (44) in L. monocytogenes strain WRLP380, a strain which conferred 1.5-fold-enhanced tolerance to BC compared to its parent strain. Together, the findings from Bland et al. (44) and our study demonstrate that this particular amino acid change is associated with FepR loss of function, and further investigation is warranted to uncover the mechanism responsible for this observed phenotype. Additionally, these findings highlight the ability of L. monocytogenes and *L. innocua* to acquire BC tolerance through the same nonsynonymous mutation in *fepR*, further emphasizing the validity of *L. innocua* as an index organism for the presence of L. monocytogenes in food production environments where BC is used for sanitation (9). Interestingly, however, no *fepR* mutations were present in parent strains of L. monocytogenes and *Listeria* spp. in this study, suggesting selection against the loss-of-function mutation in *fepR* in more complex environments. Hence, the practical implications of *fepR* mutations in natural environments remains to be determined.

### The BC tolerance phenotype is similar across L. monocytogenes and *Listeria* spp. with efflux mechanisms mediated by *bcrABC* and *qacH* or *fepR* mutations.

The presence of *bcrABC* and *qacH* in strains of L. monocytogenes and *Listeria* spp. from this study was associated with higher phenotypic tolerance to low levels of BC (parent MICs of 4 to 6 mg/L) than that of strains of L. monocytogenes and *Listeria* spp. that did not carry these genes (parent MICs of 1 to 2 mg/L). Similar findings have been reported for isolates carrying *bcrABC* and *qacH* obtained from a variety of food processing environments (17, 25, 27). For example, one study (17) reported that L. monocytogenes isolates carrying *qacH* showed MICs ranging from 5 to 12 mg/L (compared to strains without *qacH* that showed MICs of ≤ 5 mg/L), and Cooper et al. (25) showed that MICs for L. monocytogenes isolates carrying *bcrABC* were 2.5- to 3.5-fold higher than L. monocytogenes isolates that did not possess a BC resistance gene.

Notably, none of the adapted isolates in our study that were derived from parent strains carrying *bcrABC* or *qacH* acquired a mutation in *fepR*. Moreover, no L. monocytogenes or *Listeria* spp. with any of these three genotypes associated with BC tolerance (presence of *bcrABC* or *qacH* or nonsynonymous mutation in *fepR*) showed adapted MICs of >6 mg/L. Because there does not seem to be any phenotypic advantage in having one of these three genotypes over the others, we hypothesize that there was a lack of selective pressure for strains of L. monocytogenes and *Listeria* spp. already carrying either *bcrABC* or *qacH* to acquire inheritable tolerance through nonsynonymous mutations in *fepR*.

### The tolerance of L. monocytogenes and *Listeria* spp. to low levels of BC does not correlate with increased survival in use levels of BC.

Overall, the majority of isolates of L. monocytogenes and *Listeria* spp. in this study were able to acquire inherited tolerance to low levels of BC at a biological barrier of 6 mg/L. However, when we compared the survival of adapted isolates with acquired tolerance to low levels of BC to that of their respective parent strains at a use-level concentration of 300 mg/L BC, the adapted isolates did not show better survival than their parent strains. These findings are in accordance with those of Kastbjerg and Gram (48), who found that L. monocytogenes strains that were adapted to grow in 48 mg/L of BC did not show better survival in 125 mg/L BC than L. monocytogenes parent strains.

Additionally, the 11 parent strains carrying *bcrABC* and *qacH* also did not show increased survival after exposure to 300 mg/L BC compared to the other 56 parent strains in this study. In a study by Cooper et al. (25), the authors suggest that the presence of BC resistance genes, specifically *bcrABC*, can represent indicators for L. monocytogenes persistence in food processing environments. Based on our results, while the presence of *bcrABC* confers tolerance of L. monocytogenes and *Listeria* spp. to BC at levels below use-level concentrations, *bcrABC* does not confer increased survival of L. monocytogenes and *Listeria* spp. at use-level concentrations of BC. Therefore, based on our results, it is unlikely that the presence of *bcrABC* represents a plausible indicator of persistence of L. monocytogenes and *Listeria* spp. in food processing environments.

### Conclusions.

Overall, our results demonstrate that nearly all strains of L. monocytogenes and *Listeria* spp. tested here showed the capacity to acquire inheritable tolerance to low levels of BC, presumably through nonsynonymous mutations in *fepR*. This level of tolerance associated with nonsynonymous mutations in *fepR* was comparable to the level of tolerance observed in L. monocytogenes and *Listeria* spp. that carry resistance genes *bcrABC* and *qacH*. In addition, our results showed that strains of L. monocytogenes and *Listeria* spp. that acquired tolerance to low levels of BC did not show significantly enhanced survival when exposed to concentrations of BC recommended for sanitation of food contact surfaces in food processing environments. These findings provide evidence to support that BC applied to food contact surfaces at typical use-level concentrations for quaternary ammonium compounds (e.g., those outlined in U.S. guidance documents, i.e., 21 CFR 178.1010 [13]) will have similar effectiveness at controlling L. monocytogenes and *Listeria* spp., regardless of whether the strains of L. monocytogenes and *Listeria* spp. are tolerant to low levels of BC. Additional studies are needed to explore the importance of horizontal gene transfer of BC resistance genes (e.g., *bcrABC*, *qacH*) in populations of L. monocytogenes and *Listeria* spp. under different conditions (e.g., in multispecies biofilms exposed to BC) to assess the emergence of strains of L. monocytogenes and *Listeria* spp. with enhanced survival to use-level concentrations of quaternary ammonium compounds.

## MATERIALS AND METHODS

### Bacterial isolates and culture preparation.

For this study, a diverse set of 67 strains of L. monocytogenes and *Listeria* spp. from different fresh produce-associated sources was assembled. The selected strains represent preharvest (*n* = 9), postharvest (*n* = 49), and retail (*n* = 9) sources associated with fresh produce, including environmental samples (e.g., soil, water, environmental swabs) as well as actual produce samples (Table 1). The 67 strains were selected from a larger collection of 588 produce strains (33), which had been characterized by whole-genome sequencing and initial screening for inactivation when exposed to use-level concentrations of three sanitizers, including BC. These data were used to select diverse BC-tolerant and -sensitive strains in this study. Strains were initially stratified into the 10% most BC-tolerant and -sensitive strains based on the preliminary data. For L. monocytogenes, we also selected nine of the most prevalent clonal complexes (CCs) within the entire isolate collection, as well as the three most common CCs associated with lineage III (to ensure representation of lineage III, which is less frequent than lineages I and II), yielding 12 common CCs. For these 12 CCs, we identified strains that represented the top 10% most BC-tolerant and -sensitive strains where available. For CCs that did not include strains that represented the 10% most BC-tolerant and -sensitive strains, we randomly selected strains for inclusion. In addition, the top three most tolerant isolates and top three most sensitive isolates from the entire culture collection were also selected if not already included in the strain set. Finally, any L. monocytogenes strains that represented unique genotypes with regard to presence/absence of *bcrABC* and *qacH* were also included (e.g., we included strain FSL R12-0189, which carried *qacH* but was not classified in the top 10% most tolerant or sensitive strains). With this strategy, the final L. monocytogenes set included 28 strains that represented lineages I, II, and III (10, 15, and 3 strains, respectively) and 16 different clonal groups, including 15 CCs (CC4, CC5, CC6, CC9, CC19, CC29, CC37, CC155, CC193, CC204, CC268, CC369, CC388, CC434, CC1789), and one singleton (ST1861); a singleton refers to a clonal group that has a sequence type (ST) that differs from all other existing STs by at least two alleles (49). In this set, 12 of the strains represented the 10% most BC-tolerant strains, 11 of the strains represented the 10% most BC-sensitive strains, and 5 of the strains did not fall into these categories but were selected because they represented either lineage III (*n* = 3) or a unique genotype with regard to the presence of *bcrABC* or *qacH* (*n* = 2). For species of *Listeria* other than L. monocytogenes (i.e., *L. innocua*, *L. ivanovii*, *L. marthii*, *L. seeligeri*, *L. welshimeri*), we picked the top 10% most BC-tolerant and -sensitive strains within each species. In addition, we included one *L. welshimeri* isolate that carried *bcrABC* and a single *L. marthii* isolate that contained the gene *lmo1409*, which represented a unique genotype for *L. marthii* strains from the original culture collection. The final strain set for non-*monocytogenes Listeria* spp. included 12 *L. innocua*, two *L. ivanovii*, three *L. marthii*, 14 *L. seeligeri*, and eight *L. welshimeri* strains (Table 1). More information about the strains, including available metadata, serotypes, and associated publications, can be found on the Food Microbe Tracker (https://www.foodmicrobetracker.net/login/login.aspx) under a strain’s isolate ID (e.g., FSL H9-0078) (Table 1) (50).

Strains were streaked from −80°C freezer stocks (frozen in brain heart infusion [BHI] broth with 15% glycerol) onto brain heart infusion agar (BHIA; BD, Franklin Lakes, NJ), followed by incubation at 37°C for 24 h. Plates were stored at 4°C for at least 24 h and up to 7 days for use in experiments. To prepare bacterial cultures, 5 mL of BHI broth was inoculated with a single colony, followed by static incubation at 22°C for 34 to 36 h. This culture was diluted 1:1,000 into fresh BHI broth, followed by static incubation at 22°C for 34 to 36 h to yield stationary-phase bacterial cultures (as determined through preliminary experiments; data not shown). Bacterial cultures grown to stationary phase consistently showed initial levels between 8.5 and 9.2 log CFU/mL.

### MIC assay.

MICs of BC for individual strains were determined with the broth microdilution method, performed according to CLSI guidelines (CLSI standard M07-A9), with slight modifications in endpoint determinations (51). Briefly, BHI broth was supplemented with BC (*n*-alkyl dimethyl benzyl ammonium chloride; Sigma-Aldrich, St. Louis, MO; CAS no. 63449-41-2) to achieve appropriate BC target concentrations. For the MIC assays, bacterial cultures grown to stationary phase as described above were diluted 1:1,000 and inoculated into BHI broth supplemented with BC (BHI-BC) to yield final BC concentrations of 0.25, 0.5, 1, 2, 4, 6, 8, 10, and 12 mg/L and a final bacterial concentration of ~10^6^ CFU/mL per well. Individual strains were also inoculated into BHI broth without BC to serve as positive growth controls, and all concentrations of BHI-BC without bacterial inoculation were included to serve as negative controls for each MIC assay. Absorbance (optical density) of cultures was measured at 600 nm (OD_600_) in a microplate reader (Biotek Instruments, Winooski, VT) before (T0) and after incubation for 24 h at 22°C (T24). Differences in absorbance before and after 24 h of incubation (ΔOD_600_) were calculated, and values of >0.100 indicated strain growth at a given BC concentration. This endpoint determination method, which deviates from the method recommended in the broth microdilution method (51), was used to account for any changes in absorbance that may occur immediately following inoculation of BHI-BC with cultures of L. monocytogenes or *Listeria* spp. at T0. No subsequent confirmation of growth of L. monocytogenes and *Listeria* spp. by using culture-based methods was performed, in accordance with the procedures outlined in the broth microdilution method (51).

### Serial passage experiments.

Serial passage experiments were performed to assess the capacity of L. monocytogenes and *Listeria* spp. to acquire enhanced tolerance to BC. For serial passage experiments, each individual *Listeria* strain was serially passaged in increasing concentrations of BHI-BC in a 96-well microtiter plate with a 200-μL volume. Bacterial cultures in stationary phase were diluted to a final concentration of ~10^6^ CFU/mL at an initial concentration of 0.25 mg/L BHI-BC, followed by static incubation for up to 48 h at 22°C to allow for growth. Growth was assessed by measuring ΔOD_600_ at 24 h, and cultures were incubated for another 24 h if they did not show growth after 24 h. When growth was detected, cultures were subcultured 1:10 in the next incremental concentration of BHI-BC (i.e., 0.5, 1, 2 mg/L, with subsequent incremental increases of 2 mg/L BHI-BC). When a strain failed to show growth in a given BC concentration, a 100-μL aliquot from the well in which the strain failed to show growth was diluted in phosphate-buffered saline (PBS). Dilutions were then plated on BHIA and incubated for 24 h at 37°C for enumeration. The BC concentration at which strains failed to show growth was recorded as the “BC passaged MIC.”

After serial passaging and plating onto BHIA, strains were assessed for the stability of their acquired tolerance to BC. Two individual colonies from enumeration plates (defined as adapted isolate A and adapted isolate B) from the serial passage experiment were substreaked onto BHIA in the absence of BC and incubated at 37°C for 24 h. This substreaking procedure was repeated for a total of seven times, and individual colonies of adapted isolate A and adapted isolate B from the seventh substreaked plate were assessed for their “adapted MIC” as detailed above. The same colonies of adapted isolate A and adapted isolate B used for MIC experiments were frozen in BHI broth with 15% glycerol and stored at −80°C.

### WGS and bioinformatics analysis.

Whole-genome sequencing (WGS) was performed on 16 adapted isolates. The resulting sequences were compared to sequences of parent strains to evaluate the presence of genetic mutations. For selection of isolates for WGS, all adapted isolate A isolates were initially screened to include only isolates that displayed an adapted MIC at least 2-fold higher than that of their respective parent MIC. From this set, 16 adapted isolate A isolates were randomly selected for WGS characterization. Single colonies of adapted isolate A isolates were inoculated into 5 mL BHI broth and incubated for 15 to 18 h at 37°C, and then DNA from cultures was extracted using a QiAamp DNA minikit (Qiagen, Germantown, MD) in accordance with the manufacturer’s instructions. Genomic DNA was sequenced using the Illumina NextSeq 500 platform (Illumina, Inc., San Diego, CA) with 2 × 150 bp paired-end reads. The software Trimmomatic v0.39 was used to trim adapters and low-quality bases from raw sequencing data, and quality assessment was performed using FastQC v0.11.8 (https://www.bioinformatics.babraham.ac.uk/projects/fastqc) (52). Reads were assembled using SPAdes v3.15.2 using careful mode. Quality control of assemblies was performed using QUAST v5.0.2 (53), and average coverage was determined using SAMtools v1.11 (54). Genomes with an average coverage greater than 40× were included in genomic analysis. Contigs smaller than 500 bp were removed.

The genomes of adapted isolates were compared with their respective parental genomes through high-quality single nucleotide polymorphism analysis (hqSNP) using the CFSAN SNP Pipeline v2.2.1 (55). For each parent strain-adapted isolate pair, the CFSAN SNP Pipeline was run twice, once aligning the paired-end reads of the adapted isolate to the complete genome assembly of the parent strain and once aligning the paired-end reads of the parent strain to the complete genome assembly of the adapted isolate. SNPs that were identified in both analyses represented reliably identified SNP differences and were reported. If SNPs were located in an open reading frame, the sequence of the open reading frame was searched in NCBI against the nonredundant protein sequences (nr) database using the BLASTX program to determine gene identities and/or their putative associated protein functions (56). SNPs were classified as synonymous (i.e., silent mutations) or nonsynonymous (i.e., missense, nonsense, or frameshift mutations) in Geneious Prime v2021.1.1.

### PCR and Sanger sequencing.

Mutations in *fepR* in adapted isolates were identified through PCR amplification, followed by Sanger sequencing of *fepR*. For lysate preparation, individual colonies of adapted isolate A isolates were resuspended in 100 μL distilled H_2_O, and DNA was extracted by heat lysis via incubation at 95°C for 15 min. After incubation, the suspension was centrifuged at 14,000 × *g* for 10 min, and supernatant was used as a template for PCR. Two conventional PCR assays were developed and performed using *de novo*-designed primers (Table 6) for amplification of 886-bp (for *L. innocua*, *L. marthii*, and L. monocytogenes isolates) and 819-bp (for *L. seeligeri* and *L. welshimeri* isolates) PCR products that contained the sequence of *fepR* (585 bp). PCR was conducted on an ABI 2720 thermal cycler (Thermo Fisher), using GoTaq green master mix (Promega) with an initial denaturation of 5 min at 95°C, followed by 30 cycles of denaturation for 20 s at 95°C, annealing for 30 s at 55°C, and extension for 1 min at 72°C, with a final extension of 10 min at 72°C. The remaining primers and deoxynucleoside triphosphates (dNTPs) were digested by adding exonuclease I (10 U; Thermo Fisher) and shrimp alkaline phosphatase (1 U; Thermo Fisher) to PCR products and incubating the samples at 37°C for 45 min, followed by 80°C for 15 min. Sanger sequencing was performed on PCR products by the Biotechnology Resource Center (Cornell University, Ithaca, NY). Alignments of *fepR* for each parent strain and its corresponding adapted isolate A were performed in Geneious Prime v2021.1.1.

**TABLE 6:** Primers for *fepR* in *Listeria* strains used in this study

Target organisms	Primer	Sequence (5′ to 3′)^*a*^	Reference
*L. marthii, L. innocua,* L. monocytogenes	*fepR*-mim-F	ACGAATTGATTAGCGARTTTTTAGAAGAAA	This study
	*fepR*-mim-R	GCCKAGCTTATGCCCAACA	This study
*L. seeligeri, L. welshimeri*	*fepR*-sw-F	ATMACTTACGAACGGTCGTCAGTT	This study
	*fepR*-sw-R	GTACGGCWATATTTATCCCAGCAAG	This study	

a Sequences contain degenerate sites to compensate for variability in target sequence. R means A or G, K means G or T, M means A or C, and W means A or T.

PCR amplification of *bcrABC* and *qacH* in parent strains and adapted isolates was performed using primers and conditions described previously (24).

### Inactivation of parent strains and adapted isolates of L. monocytogenes and *Listeria* spp. at a use-level concentration of BC.

To determine whether adapted isolates showed increased survival (compared to parent strains) at use-level concentrations of BC, all 67 parent strains and all 67 adapted isolate A isolates were exposed to a use-level concentration of BC for 30 s. Prior to BC exposure, 1 mL of each bacterial culture was centrifuged at 10,000 × *g* for 2 min to pellet cells. Pellets were resuspended in 1 mL of phosphate-buffered saline adjusted to pH 8.0 (PBS-pH 8.0), and 200-μL aliquots of cell suspensions were subsequently transferred into individual wells of a 96-well deep-well plate. Then, 200 μL of 600 mg/L BC (dissolved in PBS-pH 8.0) was added to each cell suspension, yielding a final exposure level of 300 mg/L BC. Cells were exposed to this BC concentration for a total of 30 s, with pipetting up and down 10 times throughout the exposure period. Control cell suspensions were exposed to 200 μL PBS-pH 8.0 for 30 s. For neutralization, 400 μL of 1.43× Dey-Engley neutralizing broth (D/E broth; BD) was added by pipetting up and down 10 times, followed by incubation for 5 min at room temperature (~20 to 22°C). Neutralized cultures were serially diluted in D/E broth, followed by plating onto BHIA, incubation for 24 h at 37°C, and enumeration of colonies. Bacterial log reductions were calculated by subtracting the log CFU/mL of control cultures from log CFU/mL of BC-treated cultures.

### Coculture experiments.

For coculture experiments, seven L. monocytogenes strains were selected (using no formal randomization procedures or prescreening criteria) from the set of 28 L. monocytogenes strains used in monoculture experiments. The focus of the selection was on strains with existing BC resistance genes to probe whether rearrangements of these genes could give rise to adapted isolates with enhanced tolerance to BC. The final L. monocytogenes strain set included five strains that carried *bcrABC*, one strain that carried *qacH*, and one strain that did not carry a BC resistance gene (Table 5). For each coculture experiment, four strains of L. monocytogenes were grown separately to stationary phase in BHI broth as described above, followed by combining 1 mL of the stationary-phase culture of each strain into a tube (yielding 4 mL of culture with about 9 log CFU/mL of each strain), thorough vortexing, and subsequent centrifugation for 10 min at 4,000 × *g*. Each coculture cell pellet was resuspended in 200 μL BHI broth, and the resuspension was transferred onto a 0.45-μm filter placed on a BHIA plate, followed by static incubation for 48 h at 22°C; this filter mating protocol has previously been detailed (30) and was used to facilitate horizontal transfer of resistance genes. As a control, monocultures of each of the seven individual strains were plated in a 200-μL volume on a 0.45-μm filter placed on BHIA and incubated for 48 h at 22°C. After incubation, each filter (including those for the monoculture controls) was removed with forceps, transferred into a 50-mL conical vial, and washed with 5 mL BHI broth by pulse vortexing. Filters were aseptically removed, and filter rinsates were diluted to achieve an OD_600_ of ~0.2. OD-adjusted cultures were used to perform a MIC experiment (“filter plate MIC”) and were serially passaged in BC as described above. After serial passaging, three individual colonies from enumeration plates were substreaked seven times, and each of the three isolates was used to perform adapted MIC experiments.

### Statistical analysis.

Data were analyzed in R version 4.0.2 (R Core Team). MICs were log transformed to satisfy normality assumptions. Linear mixed-effects models were fit using the lme4 package to determine (i) the effect of the interaction between the type of MIC (i.e., parent, BC passaged, adapted), and whether an isolate was L. monocytogenes or *Listeria* spp. (i.e., *L. innocua*, *L. ivanovii*, *L. marthii*, *L. seeligeri*, or *L. welshimeri*), on the outcome of MIC value, (ii) the effect of the interaction between the type of MIC (i.e., parent, BC passaged, adapted), and whether an isolate carried a BC resistance gene (*bcrABC* or *qacH*), on the outcome of MIC value, (iii) the effect of whether an adapted isolate showed a mutation in *fepR* on the outcome of MIC value, and (iv) the effect of the type of MIC (i.e., filter plate, BC passaged, adapted), and whether L. monocytogenes strains were grown in cocultures or monocultures, on the outcome of MIC value. For these analyses, type of MIC, L. monocytogenes versus *Listeria* spp., and BC resistance gene (presence or absence) were considered fixed effects, and each individual isolate’s FSL ID was considered a random effect. Analysis of variance (ANOVA) was performed on linear mixed models, followed by *post hoc* analysis of estimated marginal means with Tukey’s adjustment using the emmeans package in R (57).

To assess the effect of exposure to use-level concentrations of BC on the log reductions of parent strains compared to adapted isolates, an unpaired *t* test was used to compare the mean log reductions of all parent strains to the mean log reductions of all adapted isolates. One-way ANOVA was performed to test for the effect of presence of a BC resistance gene on the outcome of log reduction following exposure to use-level concentrations of BC. *P* values of ≤0.05 were considered statistically significant. The detection limit for bacterial colony enumeration was 2 log CFU/mL. In cases where no colonies were observed, the value for the limit of detection (2 log CFU/mL) was used for statistical analyses.

### Data availability.

Whole-genome sequence data related to this study are available in the NCBI Sequence Read Archive (SRA) under BioProject accession number PRJNA761983. All raw data files and R code used to carry out statistical analysis are available on GitHub (https://github.com/sjb375/Listeria_BC_adaptation).
